# Neutropenia and infectious events during off-label treatment with venetoclax in children with malignant disease: a pharmacovigilance analysis of FDA adverse event reporting system reports

**DOI:** 10.1007/s00277-026-06951-z

**Published:** 2026-04-24

**Authors:** Edoardo Muratore, Valentina Giunchi, Michele Fusaroli, Francesco Baccelli, Francesca Gottardi, Elisabetta Poluzzi, Riccardo Masetti, Emanuel Raschi

**Affiliations:** 1 Pediatric Hematology and Oncology, IRCCS Azienda Ospedaliero-Universitaria di Bologna, Bologna, 40138 Italy; 2 Department of Medical and Surgical Sciences, University of Bologna, Bologna, Italy

**Keywords:** Venetoclax, Children, Pediatric hematology, Acute leukemia, Pharmacovigilance

## Abstract

Venetoclax, a BCL-2 inhibitor, is increasingly used off-label in pediatric malignancies, but safety data remain limited. This pharmacovigilance study analyzed 488 pediatric reports from the FDA Adverse Event Reporting System (FAERS) involving neutropenia and infection with venetoclax use. Disproportionately reported events included sepsis, leukopenia/agranulocytosis, and pseudomembranous colitis. Most cases involved acute myeloid leukemia or myelodysplastic syndromes and combination therapy. Network analysis revealed co-reporting of hematologic and infectious toxicities. These findings emphasize the need for vigilant monitoring and dedicated pediatric studies to guide venetoclax use and improve supportive care strategies in this vulnerable population receiving intensive or multimodal treatments.

**Supplementary Information:**

The online version contains supplementary material available at 10.1007/s00277-026-06951-z.

## Introduction

Venetoclax, a selective oral inhibitor of the anti-apoptotic protein B-cell lymphoma 2 (BCL-2), has revolutionized the treatment landscape of several adult hematologic malignancies, including chronic lymphocytic leukemia and acute myeloid leukemia (AML) [1]. In recent years, its use has extended beyond approved adult indications, with growing interest in its off-label application in pediatric patients with relapsed or refractory leukemias and other high-risk malignant diseases [2, 3]. Early clinical experiences suggest promising efficacy in this setting [4, 5]; however, comprehensive safety data in children remain sparse, but urgently needed to promote appropriate use.

One of the most concerning aspects of venetoclax therapy is its myelosuppressive potential, particularly neutropenia and the associated risk of severe infection, a complication that may be of increased concern in pediatric populations undergoing intensive, multimodal treatment regimens [6]. As venetoclax use becomes more widespread in children in heterogeneous clinical settings, especially outside the context of clinical trials, it becomes essential to leverage post-marketing safety data to guide supportive care strategies. In this pharmacovigilance study, we described neutropenia and infectious events reported with venetoclax use in children with malignant disease using a public global adverse event reporting database.

## Methods

We used data from the Food and Drug Administration Adverse Event Reporting System (FAERS) [7] through the DiAna R package [8] up to March 31, 2024. Cases of interest were reports including venetoclax as suspect, neutropenia and/or infection as adverse events, and an oncologic disease, in patients under 18 years (Table S1).

We performed a descriptive analysis of demographic clinical, and reporting features. Furthermore, we described transformed variables, including whether venetoclax was taken as monotherapy or in combination (Table S2), co-reporting with triazole antifungals requiring a priori venetoclax dose adjustment [9] (Table S2), and the specific oncologic indication. The ideal dose was calculated based both on the weight of the patients according to a weight-adjusted adult dose of 400 mg [10], and on the body surface according to the recommended phase II dose of 360 mg/m2 [11], and compared to actual dosages accounting age and dose information. Both dosage standards were applied due to the lack of consensus in the literature regarding pediatric dosing regimens.

To identify whether neutropenia and infectious events (Table S1) was disproportionally reported with venetoclax, we calculated the Information Component (IC) [12], an established Bayesian disproportionality approach, using as a comparator both the oncologic (RG1) and the leukemic (RG2) populations, to account for confounding by indication (subjects with malignancies, especially leukemias, are more susceptible to infections). The threshold for significance (disproportionality signal, indicating a higher-than-expected reporting) was defined by a lower limit of the 95% credibility interval > 0.

Reports characteristics and disproportionalities of children cases were also compared with the corresponding adult cases [13, 14], stratified by age group (young adults 18–39, middle-aged adults 40–64, and older adults > 64).

To investigate co-reported events, a network analysis using the Ising approach [15–17] was conducted, with the aim of gaining insights into potential syndromes.

## Results

The pediatric population with neutropenia and infectious-related events following venetoclax use consisted of 488 reports (Table 1). The majority involved males (55%), with acute myeloid leukemia or myelodysplastic syndromes (AML/MDS, 62% vs. 51% in young adults). Hospitalization and death were reported in 48% and 24% of cases, respectively. In most cases, venetoclax was used in combination with chemotherapy (63%). Some cases were treated with higher doses than the recommended levels as explained in the methods section: 32% when considering dose per square meter and 12.5% when considering dose per kilogram. No disproportionality signals were observed in these cases. Azoles were recorded in 7% of the cases.

As with the pediatric cases, AML/MDS was the most common indication in young adults (51%), whereas indications different from AML/MDS or acute lymphoblastic leukemia/lymphoma (ALL/LYMPHOMA) were more frequent in adults (63%) and older adults (52%).

**Fig. 1:**
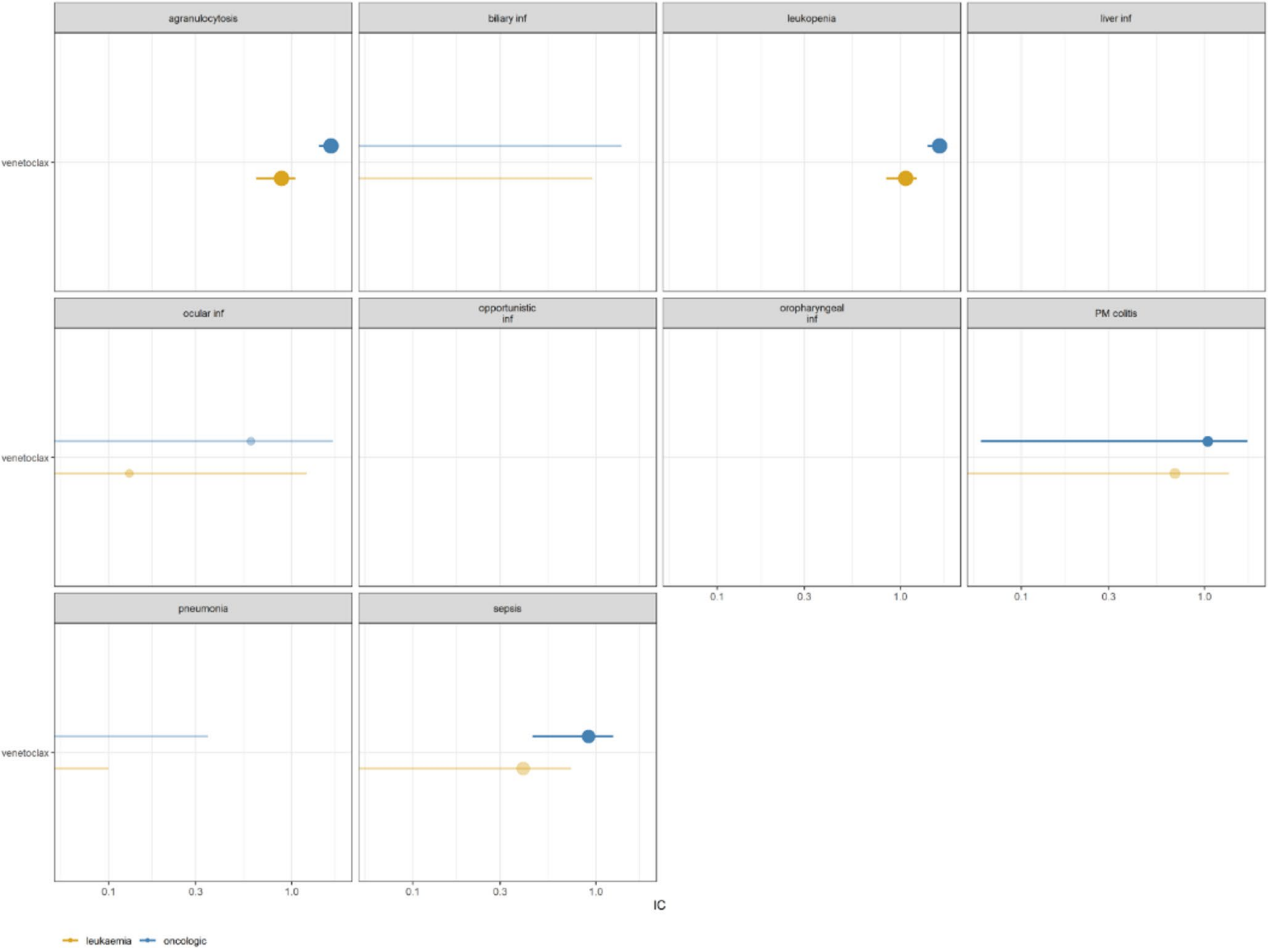
Disproportionality analysis (IC) within the two populations of interest. Only events with at least one disproportionality signal have been selected for subsequent analyses. Transparency in the line and in the dot means that the IC is not statistically significant

The events disproportionally reported with venetoclax administration in children included sepsis (IC = 0.71 [0.16–1.1] within RG1, *N* = 51) leukopenia (1.69 [1.44–1.87] within RG1, 1.12 [0.87–1.3] within RG2), agranulocytosis (1.74 [1.49–1.93] within RG1, 0.98 [0.72–1.16] within RG2), grouped in leukopenia + agranulocytosis (*N* = 214) in the following analysis, and pseudomembranous colitis (PM colitis) (1.3 [0.32–1.97] within RG1, 1 [0.02–1.66] within RG2, *N* = 12) (Fig. 1). AML/MDS was the most common indication in patients experiencing sepsis, while indications different from AML/MDS or ALL/LYMPHOMA were more frequently reported in the leukopenia and/or agranulocytosis group, as well as in the PM colitis group (Table S3). Conversely, no neutropenia and infectious-related events were disproportionally reported among adults (Figure S1).

Network analysis revealed distinct clusters of co-reported adverse events. Hematologic abnormalities, including cytopenias, were frequently co-reported together with elevated liver enzymes, while infectious complications such as sepsis and respiratory infections emerged as separate but interconnected clusters. Smaller clusters related to gastrointestinal symptoms, fluid retention, and neurologic complaints were also identified (Figure S2).

## Discussion

In the 488 patients with neutropenia and infection-related adverse events related with off-label venetoclax therapy, the events reported higher than expected were sepsis, agranulocytosis/leukopenia, and PM colitis, consistent with the known mechanism of action of venetoclax and aligning with the most frequent adverse events reported in the small published pediatric cohorts. The emerging association (disproportionality signal) with reported PM colitis is based on a limited number of cases and should therefore be confirmed by subsequent analytical studies considering the hypothesis-generating nature of disproportionality analysis; it may be attributable to increased antibiotic exposure associated with neutropenia and bacterial infections.

Adverse events varied by cancer type, with sepsis more common in AML/MDS and leukopenia or PM colitis in other malignancies. This likely reflects differences in treatment intensity and patient intrinsic vulnerability. Although no reporting difference emerged between venetoclax-azacitidine and venetoclax-chemotherapy, patients receiving these different combinations are likely to differ in risk profiles. Indeed, the venetoclax-chemotherapy combination was more reported than the venetoclax-azacitidine one, highlighting the more widespread use of this type of therapy in the pediatric population. For instance, prescribers may prefer venetoclax–azacitidine in less fit AML patients as a bridge to transplant or in previously untreated children with MDS. However, disproportionality analysis can only partially account for confounding, particularly co-administered therapies, due to the limited information captured in adverse event reports; dedicated pediatric studies investigating venetoclax in combination with non-cytotoxic therapies in different settings are needed to infer causality.

Moreover, no association was found between these adverse events and either off-range dosing or co-administration with azoles. Despite the aforementioned limitations of this pharmacovigilance analysis, our finding underscores the need for dedicated pharmacological studies to better characterize venetoclax pharmacokinetics and pharmacodynamics in pediatric patients [18].

Our study highlights the value of FAERS in monitoring off-label drug safety, with pediatric reports often more detailed than adult ones, especially with regard to weight, enabling robust analysis for signal detection and validation (although not to estimate risk). Information in these fields is often missing from adverse event reports. Additionally, we could impute missing dosing metrics, showing how informed data processing can enhance signal detection despite known limitations.

No disproportionality signals related to neutropenia or infection were identified in the adult cohort as a whole and in any subgroup. This discrepancy could reflect differences in reporting behaviors or therapeutic combinations [for instance, adult patients have received less myelosuppressive co-treatments (Table 1)], although other reporting biases such as dilution effect, channeling bias and age-specific under-reporting in adults cannot be ruled out. Also, we cannot exclude a distortion of results due to duplicate reporting.

**Table 1:** Descriptive analysis of pediatric cases and of the three groups of adults. Categorical variables are presented as absolute numbers and percentages, while continuous variables are reported as medians (IQR). Combinations in children have been categorized in the following way: if aza + chemo, chemo, if aza + monoclonal ab, aza, if aza + small molecules, aza, if chemo + small molecules, chemo, if monoclonal ab + small molecules, small molecules

Variable	Pediatric Cases (*N*, %)	Young adults (*N*, %)	Adults (*N*, %)	Older adults (*N*, %)
N	488	884	5541	15,231
Sex				
Female	216 (44.72)	395 (46.09)	2,063 (37.97)	5,460 (36.38)
Male	267 (55.28)	462 (53.91)	3,370 (62.03)	9,548 (63.62)
Unknown	5	27	108	223
Reporter				
Consumer	74 (15.20)	190 (21.52)	1,750 (31.69)	5,983 (39.39)
Healthcare practitioner	159 (32.65)	222 (25.14)	650 (11.77)	1,572 (10.35)
Lawyer	0 (0.00)	0 (0.00)	2 (0.04)	448 (2.95)
Other	9 (1.85)	21 (2.38)	191 (3.46)	0 (0.00)
Pharmacist	14 (2.87)	18 (2.04)	128 (2.32)	437 (2.88)
Physician	231 (47.43)	432 (48.92)	2,802 (50.73)	6,749 (44.43)
Unknown	1	1	18	42
Outcome				
Death	118 (24.18)	293 (33.14)	1,693 (30.55)	5,517 (36.22)
Life threatening	29 (5.94)	32 (3.62)	196 (3.54)	476 (3.13)
Disability	3 (0.61)	12 (1.36)	31 (0.56)	83 (0.54)
Required intervention	0 (0.00)	0 (0.00)	0 (0.00)	1 (0.01)
Hospitalization	234 (47.95)	293 (33.14)	2,074 (37.43)	5,513 (36.20)
Congenital anomaly	1 (0.20)	0 (0.00)	0 (0.00)	1 (0.01)
Other serious	84 (17.21)	229 (25.90)	1,253 (22.61)	2,925 (19.20)
Non Serious	19 (3.89)	25 (2.83)	294 (5.31)	715 (4.69)
Continent				
North America	278 (56.97)	422 (47.74)	3,006 (54.25)	9,137 (59.99)
Europe	151 (30.94)	182 (20.59)	1,433 (25.86)	3,426 (22.50)
Asia	32 (6.56)	216 (24.43)	808 (14.58)	1,886 (12.38)
South America	2 (0.41)	37 (4.19)	135 (2.44)	343 (2.25)
Oceania	24 (4.92)	25 (2.83)	148 (2.67)	402 (2.64)
Africa	1 (0.20)	2 (0.23)	11 (0.20)	36 (0.24)
Unknown	0	0	0	1
Age (years)	9 (5.00–14)	30 (22.00–36)	58 (53.00–62)	74 (70.00–79)
Weight (kgs)	24 (16.00–38)	70 (58.00–78)	76 (63.00–89)	72 (61.00–83)
Unknown	256	628	3,714	10,313
Reactions (No.)	3 (1.00–4)	2 (1.00–4)	2 (1.00–4)	2 (1.00–4)
Substances (No.)	10 (3.50–19)	5 (2.00–11)	4 (2.00–11)	5 (2.00–14)
Time to onset (days)	26 (12.00–53)	22 (12.00–77)	40 (10.00-140)	45 (11.00-168)
Unknown	256	541	2,949	7,835
Indication				
ALL/LYMPHOMA	81 (37.85)	65 (7.35)	47 (0.85)	59 (0.39)
AML/MDS	133 (62.15)	452 (51.13)	1979 (35.71)	7231 (47.47)
Unknown	274	367	3515	794
Combination		NA	NA	NA
monoclonal ab	4 (0.82)	109 (12.33)	1924 (23.35)	2271 (14.91)
azacitidine	68 (13.93)	312 (35.29)	1045 (18.86)	4021 (26.40)
chemo	307 (62.91)	256 (28.96)	754 (13.61)	1077 (7.07)
small molecules	11 (2.25)	123 (13.91)	1026 (18.52)	1659 (10.89)
monotherapy	98 (20.08)	308 (34.84)	2588 (46.71)	7815 (51.31)
Azoles	34 (6.97)	NA	NA	NA
Dose/m2 >>	71.00 (31.98)	NA	NA	NA
Dose/Kg >>	61.00 (12.50)	NA	NA	NA

*Ab* antibodies, *ALL* acute lymphoblastic leukemia, *AML* acute myeloid leukemia, *MDS* myelodysplastic syndrome, *NA* not applicable

The network analysis offered additional insights into the complexity of adverse event co-occurrence. Hematologic toxicities were frequently co-reported with hepatic enzyme abnormalities, suggesting possible overlapping toxic mechanisms or potentially reflecting the underlying contribution of chemotherapy or concomitant drugs, including triazole antifungals and other antimicrobial agents. However, an ascertainment bias is also plausible since different blood anomalies are reported together just because they are detected by concomitant tests. Smaller clusters involving gastrointestinal symptoms, fluid retention, and neurological effects may also highlight emerging toxicity to monitor in future trials.

These pharmacovigilance findings underscore the need for proactive monitoring of hematologic and infectious toxicities in children receiving venetoclax, highlighting the need for dedicated pediatric venetoclax studies with integrated safety monitoring components. While off-label use has rapidly expanded due to clinical need, controlled prospective studies will be essential to fully characterize venetoclax’s safety profile, optimize dosing, and implement evidence-based supportive care protocols for pediatric patients.

## Supplementary Information

Below is the link to the electronic supplementary material.

Supplementary Material 1

## Data Availability

The datasets used and analyzed during the current study available from the corresponding author on reasonable request.
