# Supplementary material for: Neutropenia and infectious events during off-label treatment with venetoclax in children with malignant disease: a pharmacovigilance analysis of FDA adverse event reporting system reports
Source: Ann Hematol. 2026 Apr 24;105(5):264. doi: 10.1007/s00277-026-06951-z (PMC13106237; doi:10.1007/s00277-026-06951-z)
Supplement: Supplementary file 1 — Supplementary Material 1 [file 277_2026_6951_MOESM1_ESM.docx]

**Supplementary material**

**Neutropenia and infectious events during off-label treatment with venetoclax in children with malignant disease: characterization and assessment of reports from the FDA Adverse Event Reporting System**

**Table S1** – Definition of immunodeficiency adverse events

| **MedDRA level/query** | **Term(s)** |
| --- | --- |
| SMQ | "Haematopoietic leukopenia (SMQ)", "Agranulocytosis (SMQ)", "Oropharyngeal infections (SMQ)", "Liver infections (SMQ)", "Infectious biliary disorders (SMQ)", "Sepsis (SMQ)", "Pseudomembranous colitis (SMQ)", "Opportunistic infections (SMQ)", "Ocular infections (SMQ)", "Infective pneumonia (SMQ)" |
| SOC | "infections and infestations" |
| HLGT | "immunodeficiency syndromes" |
| PT | "agranulocytosis", "febrile neutropenia", "granulocytopenia", "granulocytopenia neonatal", "leukopenia", "lymphocytopenia neonatal" ,"lymphopenia", "monocytopenia", "neutropenia", "neutropenia neonatal", "cyclic neutropenia", "autoimmune neutropenia", "benign ethnic neutropenia", "basophilopenia", "b-cell aplasia", "idiopathic cd4 lymphocytopenia" |

**Definition of the populations:**

Oncologic population: all reports involving at least one event within the MedDRA ( <https://www.meddra.org/>) SOC "neoplasms benign, malignant, and unspecified (incl cysts and polyps)" or a drug classified under the WHO-ATC (https://atcddd.fhi.no/atc_ddd_index/) class L01.

Leukemic population: reports involving either a drug indicated for conditions within the HLGT "leukaemias" or reports including venetoclax or navitoclax among the drugs.

**Table S2** – List of drugs considered in combination with venetoclax

| **Group** | **Active Ingredients** |
| --- | --- |
| **Combination with** | |
| Aza | Azacitidine, decitabine |
| Small molecules drugs | Bortezomib, ibrutinib, cobimetinib, sorafenib, ruxolitinib, midostaurin, acalabrutinib, erlotinib, quizartinib, ponatinib, imatinib, nilotinib, dasatinib, trametinib, gilteritinib, zanubrutinib, alectinib, mivavotinib, bosutinib, itacitinib, osimertinib, Larotrectinib, fedratinib, cabozantinib, pralsetinib, sunitinib, capmatinib, gefitinib, asciminib, avapritinib, pacritinib, afatinib, upadacitinib |
| Chemotherapy agents | Vincristine, bendamustine, doxorubicin, cyclophosphamide, cytarabine, methotrexate, fludarabine, etoposide, daunorubicin, cisplatin, melphalan, idarubicin |
| Monoclonal antibodies | Rituximab, obinutuzumab, polatuzumab vedotin, daratumumab, alemtuzumab, ranibizumab, denosumab, alirocumab, pembrolizumab, ofatumumab, nivolumab, adalimumab, atezolizumab, gemtuzumab, ipilimumab, vedolizumab, ocrelizumab, evolocumab, bevacizumab, elotuzumab, alemtuzumab, inotuzumab, galcanezumab, tocilizumab, trastuzumab, omalizumab, brodalumab, romosozumab, sacituzumab govitecan, reslizumab, dupilumab, satralizumab, camrelizumab, teplizumab |
| **Concomitance with** | |
| Azoles | Itraconazole, voriconazole, posaconazole, isavuconazole, oteseconazole, clotrimazole, miconazole, econazole, chlormidazole, isoconazole, tiabendazole, tioconazole, ketoconazole, sulconazole, bifonazole, oxiconazole, fenticonazole, omoconazole, sertaconazole, fluconazole, flutrimazole, eberconazole, luliconazole, efinaconazole, neticonazole, lanoconazole, bifonazole |

**Table S3** – Descriptive analysis of cases categorized by events of interest. Categorical variables are presented as absolute numbers and percentages, while continuous variables are reported as medians (IQR) [minimum–maximum]. Combinations in children have been categorized in the following way: if aza + chemo, chemo, if aza + monoclonal ab, aza, if aza + small molecules, aza, if chemo + small molecules, chemo, if monoclonal ab + small molecules, small molecules.

The following variables were already present in the FAERS: sex, age, weight, continent, number of concomitant drugs, number of adverse reactions, reaction outcome (e.g., death, hospitalization), time to onset, and reporter occupation. Instead, manually processed variables encompassed the specific event(s) of interest reported, dose per kilogram, dose per square meter (calculated from weight, age, and dose information), whether the event occurred outside the therapeutic window, whether venetoclax was taken as monotherapy or in combination (specific combinations of interest are listed in **Table S2**), concomitance with azoles (**Table S2**), and the reported indication

| **Variable** | **Sepsis (N, %)** | **Leukopenia + agranulocytosis (N, %)** | **PM colitis (N, %)** |
| --- | --- | --- | --- |
| N | 51 | 214 | 12 |
| **Sex** |  |  |  |
| Female | 13 (25.49) | 98 (45.79) | 8 (66.67) |
| Male | 38 (74.51) | 116 (54.21) | 4 (33.33) |
| **Reporter** |  |  |  |
| Consumer | 0 (0.00) | 18 (8.45) | 1 (8.33) |
| Healthcare practitioner | 13 (25.49) | 86 (40.38) | 1 (8.33) |
| Other | 0 (0.00) | 4 (1.88) | 0 (0.00) |
| Pharmacist | 2 (3.92) | 5 (2.35) | 0 (0.00 |
| Physician | 36 (70.59) | 100 (46.95) | 10 (83.33) |
| Lawyer | 0 (0.00) | 0 (0.00) | 0 (0.00 |
| Unknown |  | 1 |  |
| **Outcome** |  |  |  |
| Death | 21 (41.18) | 16 (7.48) | 0 (0.00) |
| Life threatening | 6 (11.76) | 15 (7.01) | 0 (0.00) |
| Disability | 0 (0.00) | 0 (0.00) | 0 (0.00) |
| Required intervention | 0 (0.00) | 0 (0.00) | 0 (0.00) |
| Hospitalization | 18 (35.29) | 150 (70.09) | 10 (83.33) |
| Congenital anomaly | 0 (0.00) | 1 (0.47) | 0 (0.00) |
| Other serious | 6 (11.76) | 31 (14.49) | 2 (16.67) |
| Non Serious | 0 (0.00) | 1 (0.47) | 0 (0.00) |
| **Continent** |  |  |  |
| North America | 27 (52.94) | 114 (53.27) | 2 (16.67) |
| Europe | 18 (35.29) | 72 (33.64) | 6 (50.00) |
| Asia | 6 (11.76) | 11 (5.14) | 0 (0.00) |
| South America | 0 (0.00) | 0 (0.00) | 0 (0.00) |
| Oceania | 0 (0.00) | 17 (7.94) | 4 (33.33) |
| Africa | 0 (0.00) | 0 (0.00) | 0 (0.00) |
| Unknown | 0 | 0 | 0 |
| **Age (years)** | 9 (5.00-14) [2.00-17] | 9 (6.00-12) [0.00-17] | 2 (2.00-6) [2.00-8] |
| **Weight (kgs)** | 29 (17.00-40) [11.00-85] | 23 (16.50-35) [1.00-85] | 13 (11.5-14.5) [11-18] |
| Unknown | 22 | 66 | 2 |
| **Time to onset (days)** | 15 (11.00-23) [1.00-186] | 19 (12.00-49) [0.00-240] | 41 (14.25-78) [1-579] |
| Unknown | 19 | 91 |  |
| **Indication** |  |  |  |
| ALL/LYMPHOMA | 11 (21.57) | 32 (14.95) | 0 (0.00) |
| AML/MDS | 22 (43.14) | 44 (20.56) | 1 (8.33) |
| Other | 18 (35.29) | 138 (64.49) | 11 (91.67) |
| **Combination** |  |  |  |
| monoclonal ab | 0 (0.00) | 5 (2.21) | 0 (0.00) |
| aza | 8 (15.69) | 27 (11.95) | 0 (0.00) |
| chemo | 34 (66.67) | 178 (78.76) | 12 (100.00) |
| small molecules | 4 (7.84) | 4 (1.77) | 0 (0.00) |
| monotherapy | 5 (9.8) | 12 (5.31) | 0 (0.00) |
| Azoles | 3 (5.88) | 4 (1.87) | 0 (0.00) |
| **Dose/m2 >>** | 28 (35.71) | 138 (31.88) | 10 (83.33) |
| **Dose/Kg >>** | 25 (28.00) | 133 (30.83) | 10 (83.33) |


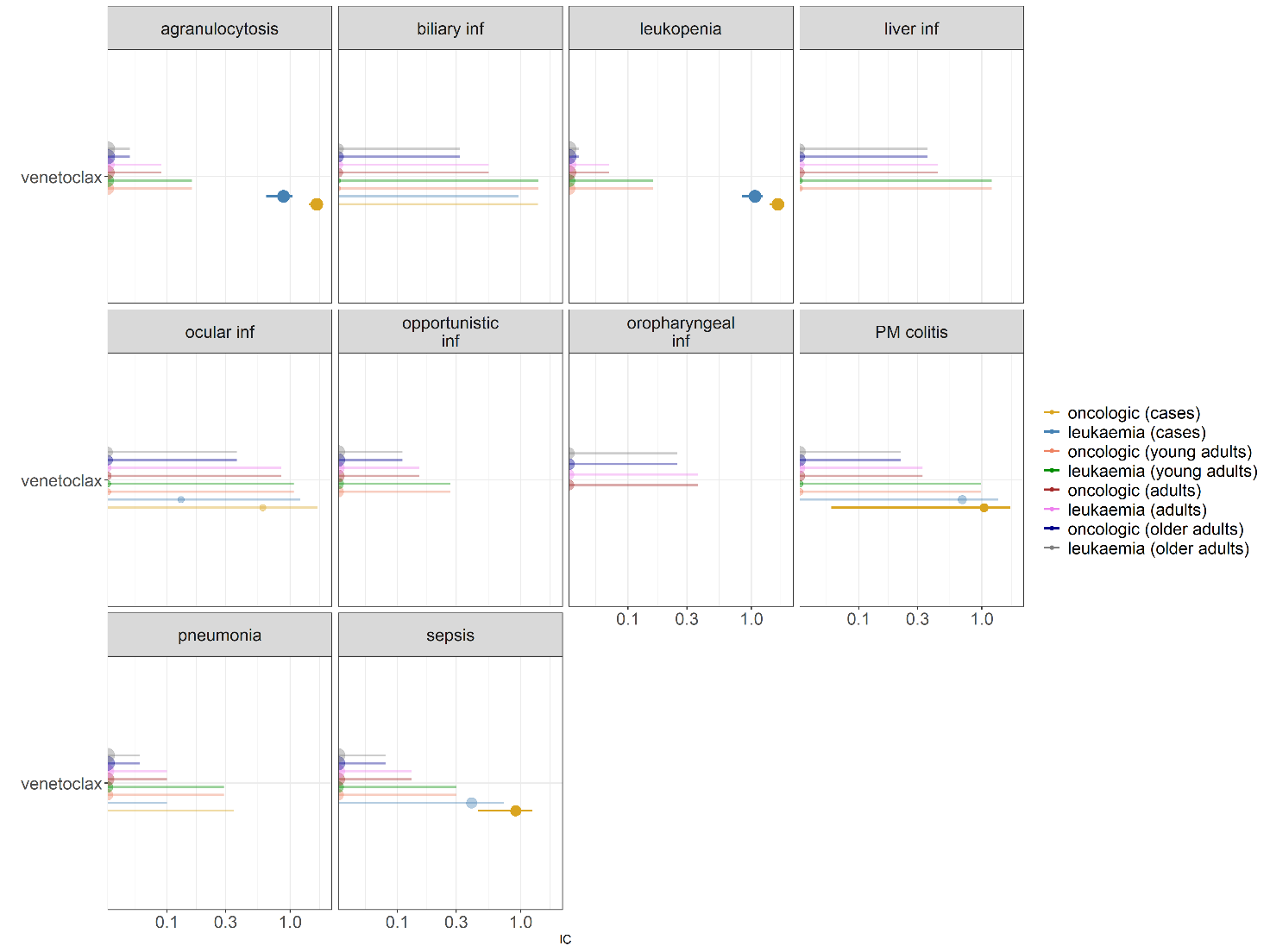


**Figure S1** - Disproportionality analysis (IC) within the oncologic and leukemic populations for cases (same as analysis in Figure S1) and for the three categories of adults (young adults, adults, and older adults).


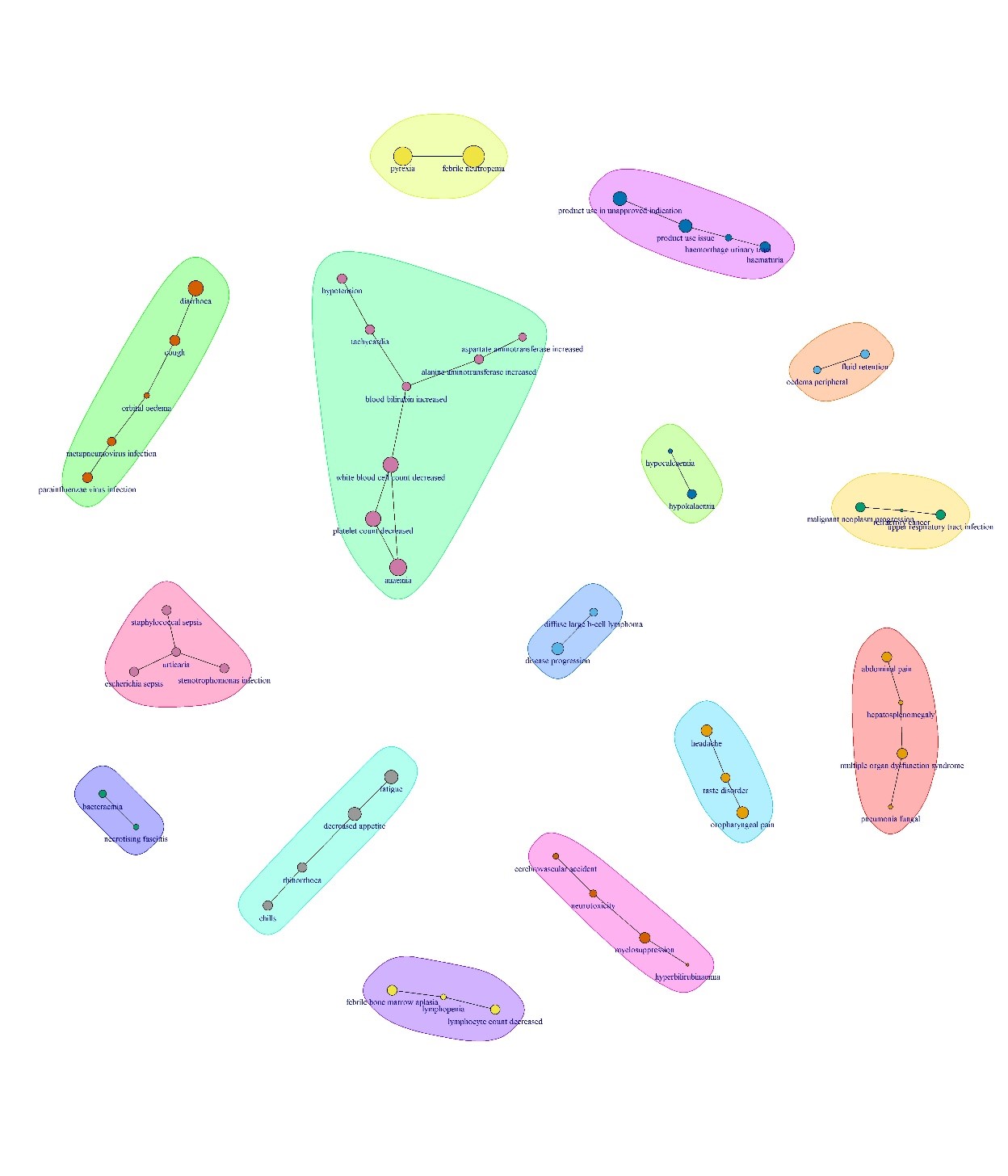


**Figure S2** – Ising network of events co-reported in cases.
